# Combination of automated sample preparation and micro-flow LC–MS for high-throughput plasma proteomics

**DOI:** 10.1186/s12014-022-09390-w

**Published:** 2023-01-07

**Authors:** Xueting Ye, Xiaozhen Cui, Luobin Zhang, Qiong Wu, Xintong Sui, An He, Xinyou Zhang, Ruilian Xu, Ruijun Tian

**Affiliations:** 1 The Second Clinical Medical College of Jinan University, the First Affiliated Hospital of Southern University of Science and Technology, Shenzhen People’s Hospital, Shenzhen, 518020 China; 2 The First Affiliated Hospital, Jinan University, Guangzhou, 510632 China; 3 Department of Chemistry and Research Center for Chemical Biology and Omics Analysis, School of Science, Southern University of Science and Technology, Shenzhen, 518055 China

**Keywords:** Plasma proteome profiling, Automated samples preparation, Micro-flow LC–MS, High throughput

## Abstract

**Background:**

Non-invasive detection of blood-based markers is a critical clinical need. Plasma has become the main sample type for clinical proteomics research because it is easy to obtain and contains measurable protein biomarkers that can reveal disease-related physiological and pathological changes. Many efforts have been made to improve the depth of its identification, while there is an increasing need to improve the throughput and reproducibility of plasma proteomics analysis in order to adapt to the clinical large-scale sample analysis.

**Methods:**

We have developed and optimized a robust plasma analysis workflow that combines an automated sample preparation platform with a micro-flow LC–MS-based detection method. The stability and reproducibility of the workflow were systematically evaluated and the workflow was applied to a proof-of-concept plasma proteome study of 30 colon cancer patients from three age groups.

**Results:**

This workflow can analyze dozens of samples simultaneously with high reproducibility. Without protein depletion and prefractionation, more than 300 protein groups can be identified in a single analysis with micro-flow LC–MS system on a Orbitrap Exploris 240 mass spectrometer, including quantification of 35 FDA approved disease markers. The quantitative precision of the entire workflow was acceptable with median CV of 9%. The preliminary proteomic analysis of colon cancer plasma from different age groups could be well separated with identification of potential colon cancer-related biomarkers.

**Conclusions:**

This workflow is suitable for the analysis of large-scale clinical plasma samples with its simple and time-saving operation, and the results demonstrate the feasibility of discovering significantly changed plasma proteins and distinguishing different patient groups.

**Supplementary Information:**

The online version contains supplementary material available at 10.1186/s12014-022-09390-w.

## Background

Plasma is the primary sample for clinical proteomics studies [1]. Although the tumor tissue provides the best opportunity for protein biomarker discovery of solid tumor, plasma also contains measurable protein biomarkers that can well reveal disease-related physiological and pathological changes [2]. Besides, plasma is easier to obtain than tissue samples, which gives an advantage for plasma-based biomarkers. Taking colon cancer, as an example, its examination and diagnosis often require invasive means such as puncture surgery for the purpose of obtaining solid tissue, which will cause damage to the patient. Although it has limited accuracy and sensitivity, the most commonly used non-invasive test in clinical practice is fecal occult blood test [3]. Based on the above considerations, current clinical diagnostic procedures are continually being improved, including blood tests such as the FDA-approved test for Septin 9 DNA methylation [4]. Several studies in recent years have successfully combined different protein markers to identify colon cancer with better discriminative capabilities [5–7]. To develop diagnostic blood biomarkers is therefore a continuous direction of clinical testing.

Mass spectrometry (MS) has become the mainstay for high-throughput proteome profiling in various biological systems. Due to the complex protein composition and the high dynamic range of proteins in plasma [1, 8], it is common to increase the depth of identification by removing high-abundance proteins and fractionating proteins or peptides [9]. However, for large-scale clinical samples, the complex sample preparation process is not only time-consuming, but even lead to technical bias [10]. Therefore, automation and integration of plasma proteomics sample preparation is a desirable approach to improve the throughput and reproducibility of clinical assays, as it can be easily standardized or scaled up. Currently, several large-scale proteomic analysis workflows for large-cohort plasma samples have been reported. Mann’s group developed the 96-well iST automated sample pretreatment platform [11] and applied it to large-cohort plasma proteome analysis [12]. The platform has also been applied to plasma proteome analysis in a variety of diseases, including liver disease [13], COVID-19 [14], etc. Recently, an automated and high-throughput solution (uHTPPP) was developed to enable large-scale plasma proteomic analysis, which automated the protocol of the Thermo Scientific EasyPep 96 MS Sample Prep Kit using a liquid handling robotic platform (Application note 65,727). We developed integrated spintip-based proteomics sample preparation technology SISPROT for processing plasma samples with convenient operation and high reproducibility. Taking advantage of its integrated two-dimensional peptide fractionation, 862 protein groups can be identified from 1 μL of plasma sample [15]. The SISPROT technology has been further improved for collecting, shipping, and processing both proteins and metabolites from dried single-drop plasma sample [16], and serum proteomic analysis of renal cell carcinoma patients [17].

In this study, we present a robust and reproducible high-throughput sample preparation workflow for micro-flow LC–MS/MS-based plasma proteome analysis. Considering the detection requirements of clinical plasma samples, we carried out in-solution digestion of plasma proteins without additional protein depletion and prefractionation. The automatic pipetting platform integrated all the in-solution sample preparation steps and could be customized according to the various sample size. Specifically, this automated workflow can handle dozens of samples at the same time in one cycle or complete the automation of large-scale samples through multiple cycles. In addition, the combination of micro-flow LC–MS/MS can achieve plasma proteome analysis with significantly improved throughput and stability. Lastly, the developed workflow was subsequently applied in a proof-of-concept plasma proteome study of colon cancer patients with different ages.

## Methods

### Sample collection

Plasma samples from 30 patients with stage III/IV colon cancer, and no other primary cancer diagnosis, including fifteen males and fifteen females (median age: 58 years, range: 30–80 years) were selected for this study from the Department of Oncology, Shenzhen People’s Hospital. Plasma samples were collected at the time of plasma biochemical examination. The clinical information of the colon cancer plasma samples has been listed (Additional file 2: Table S1). All procedures for plasma sample collection have been ethically approved by the Medical Ethics Committee of the Shenzhen People's Hospital, Shenzhen, China. Peripheral blood samples were clotted at room temperature before collecting the upper sera and stored at − 80 ℃ until further use. For method optimization of automated samples preparation, a pooled plasma sample was prepared by mixing several plasma samples. BCA assay (Thermo Fisher Scientific) was used to measure the protein concentration of each sample.

Plasma samples were prepared using the in-solution digestion protocol [12], which combined LH-1808 fully automatic liquid handling platform (AMTK, China) with optimization for plasma. The AMTK system in this study is programmed to process 32 samples simultaneously including sample predilution, protein reduction and alkylation, and digestion of proteins. The automatic process for plasma sample preparation is as follows: 5 μL of plasma sample were transferred into a 96-well plate with a 1:20 dilution by adding 95 μL of reduction-alkylation buffer which consists of 10 mM Tris (2-carboxyethyl) phosphine hydrochloride (TCEP, Sigma, Germany), 50 mM 2-Chloroacetamide (CAA, Sigma, Germany), and 50 mM Tris–HCl (pH 8, Sigma, Germany). The samples were pipetted 10 times up and down for a volume of 40 μL to mix thoroughly. A 20 μL of 20-fold diluted plasma was transferred into a new plate and heated at 95 ℃ for 15 min to denature proteins. After cooling down to room temperature, the proteolytic enzymes LysC and trypsin were mixed in (1:100 µg of enzyme to micrograms of protein, 0.6 μg of each enzyme). Digestion plate was incubated at 37 ℃ for 3 h and then quenched the reaction by 50 µL of 0.1% (v/v) trifluoroacetic acid (TFA). The obtained digested peptide samples were desalted by using home-made C18 spintip packed with 10 layers of C18 plug (3 M Empore, USA) and 3 mg of C18 beads (10 μm, Dr. Maisch GmbH, Ammerbuch, Germany) in tandem [18]. Finally, the desalted peptides were lyophilized to dryness for micro-flow LC–MS analysis.

### Micro-flow LC–MS/MS analysis

The plasma peptide samples were redissolved in 0.1% (v/v) formic acid (FA) and analyzed by using a reported micro-flow LC–MS/MS method [19]. This micro-flow LC–MS/MS system consisted of Orbitrap Exploris 240 mass spectrometer (Thermo Fisher Scientific, USA) equipped with a Dionex UltiMate 3000 RSLCnano System (Thermo Fisher Scientific, USA). The LC separation was carried out on a commercial 15 cm Acclaim PepMap 100 C18 column (1 mm i.d. × 150 mm, Thermo Fisher Scientific) at a flow rate of 50 μL/min. The buffer A used for separation was 0.1% (v/v) FA in water, the buffer B was 0.1% (v/v) FA in 80% acetonitrile. Peptides were separated with a 66 min segmented gradient as follows: 0.5% buffer B for 2 min, 0.5–6% buffer B for 0.1 min, 6–35% buffer B for 60 min, 35–90% buffer B for 0.2 min, 90% buffer B for 2 min, 90–0.5% buffer B for 0.2 min, 0.5% buffer B for 1.5 min. Full MS scans were acquired from m/z 350 to 1550 with a mass resolution of 60,000. MS/MS scans were performed in data-dependent top 12 mode. Tandem MS/MS was acquired at a resolution of 15,000 and using an isolation window of 1.3 Da. Higher energy collisional dissociation (HCD) fragmentation was set with a normalized collision energy of 30%. The dynamic exclusion time was set as 25 s.

### Data analysis

The raw data were processed using Sequest HT [20] integrated within the Proteome Discoverer (PD) software (version 2.5, Thermo Fisher Scientific) and searched against the human Uniprot FASTA database (74,811 entries, downloaded on March, 2020). The precursor and fragment mass tolerances were set to 10 ppm and 0.02 Da, respectively. A maximum of two missed cleavages was allowed. Methionine oxidation and N-terminal acetylation were set as dynamic modifications, while carbamidomethylation was applied as fixed modification. False discovery rate (FDR) of peptide spectrum matches (PSMs) and peptides were determined by searching the forward and reverse database and were validated by the Percolator algorithm at 1% based on q-values [21]. MaxLFQ algorithm integrated within MaxQuant (version 1.6.17.0) was used for the label-free quantification (LFQ) analysis of all the raw data including colon cancer plasma samples with default parameters [22]. The same human database and the same criteria were set as in the PD search. FDR based on posterior error probability (PEP) was determined by searching a reverse database and was set to 0.01 for proteins and peptides. Statistical analysis and data visualization were performed using the Perseus software (version 1.5.5.3). Common contaminants, peptides only identified by side modification and reverse were excluded for further analysis. Proteins identified from colon cancer plasma samples with at least one ‘unique + razor peptide’ and three valid values in at least one group were kept for further analysis. Proteins were subjected to a gene ontology cellular component (GOCC) enrichment analysis performed by cluster Profiler (version 3.14.3) package in R environment (version 3.6.2).

## Results

### Development of automated plasma preparation workflow

Patient plasma specimens are one of the most commonly used and easily accessible resources for clinical pathological investigation. Complicated sample preparation procedures and nano-flow LC–MS/MS-based proteomic analysis have been the mainstream methods for in-depth exploration of plasma proteomics. Conventional in-solution digestion is the most classical method can be used for protein digestion of various sample types. With the throughput requirements for clinical samples and biomarker discovery, the aim of this study is to develop and optimize the in-solution protein digestion protocol on an automated liquid handling system, achieving efficient preparation of plasma samples. The workflow of automated sample pretreatment combined with micro-flow LC–MS/MS system can be applied to the high-throughput analysis of clinical samples. The advantages of this strategy are reflected in two aspects. Firstly, compared with traditional manual analysis of only one set of samples per experiment, the liquid handling platform can process dozens of samples simultaneously in an automated manner, saving time and labor costs. Secondly, micro-flow LC–MS/MS system can significantly reduce the analysis time and improve the stability compared with nano-flow LC–MS/MS method [23].

**Fig. 1:**
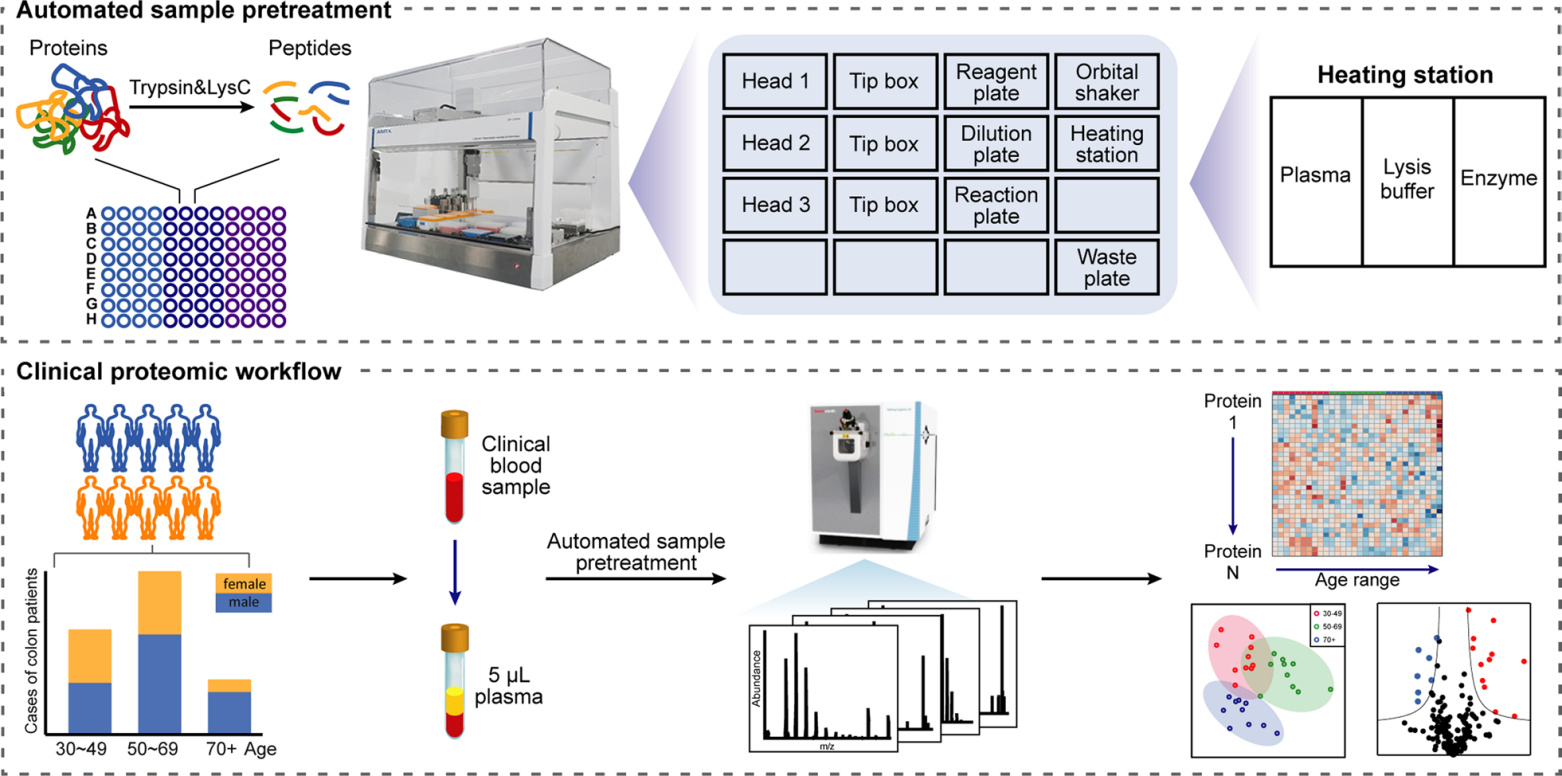
Schematic diagram of automated plasma sample preparation workflow and the application for clinical patients

In this study, we made full use of the remaining plasma samples after biochemical examination of cancer and performed all preparation steps in a 96-well plate of the AMTK-LH1808 liquid handling workstation. To evaluate the workflow, 12 of the 30 AMTK blocks in the system were selected, including functional modules such as the orbital shaker and the heating block. An overview of the deck setup is shown in Fig. 1. Mixed plasma was used as input for the protocol that we developed for automated sample preparation. The temperature adjustment range of the heating block is 4–110 ℃, and it can be set at 4 ℃ to store plasma, lysis buffer, and enzymes, temporarily. The fully automated protocol design includes an on-deck sample dilution process with a mixture of TCEP and CAA solutions to reduce and alkylate proteins. In-well trypsin and LysC digestion and termination reactions were also incorporated into the workflow, allowing high-throughput processing of plasma samples in the same microplate. Since plasma is rich in protein (~ 60 μg/μL), there is no nanogram injection volume requirement for its analysis. An online micro-flow LC–MS/MS system with a flow rate of 50 μL/min and Orbitrap Exploris 240 mass spectrometer were used to increase the analysis throughput while taking into account both identification and accurate quantification performance.

### Optimization of the automated plasma preparation workflow

**Fig. 2:**
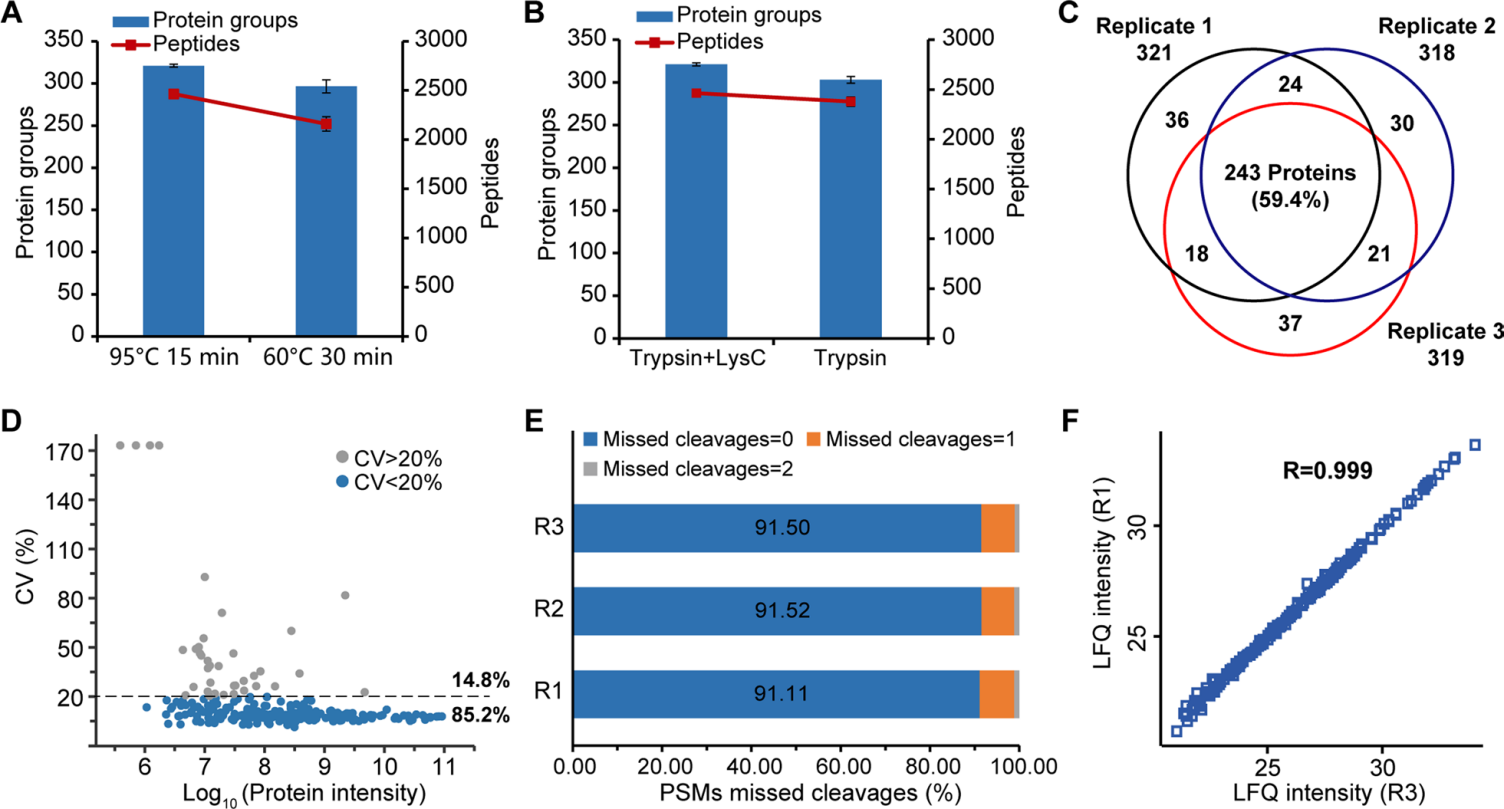
Optimization of the automated plasma sample preparation workflow. **A** Comparison of reduction and alkylation conditions for the number of identified protein groups and peptides. **B** Comparison of enzymes used in combination or alone. **C** Identification reproducibility of three batches of the same plasma sample processed at the same plate. **D** CVs of all quantified proteins were calculated for the 3 replicates. Proteins with CV < 20% are colored in blue and those with CV > 20% in gray. **E** Distribution of PSMs in each missed cleavages% levels. **F** Correlation of the protein intensities between replicate 1 and replicate 3. Correlation of protein intensities between the other replicates were appended in Figure S2

A total of two reactions occurred in the pretreatment process of plasma samples, namely reduction-alkylation reaction and protein digestion. The reaction conditions here are the main factors affecting automated sample preparation performance. Therefore, we investigated critical reaction conditions, including reaction time, temperature, and the combination of proteases. As shown in Fig. 2A, one-step reduction and alkylation were carried out at 95 ℃ for 15 min brought better results than 60 ℃ for 15 min. The error bar of the former reaction conditions was also significantly smaller than that of the latter in three repeated experiments, indicating the completeness of the reaction. In addition, digestion with trypsin in combination with LysC improved digestion efficiency compared to digestion with trypsin alone under the same duration condition (Fig. 2B). Using optimized conditions, we were able to identify more than 300 protein groups and 2400 unique peptides on average from a single analysis by using the Orbitrap Exploris 240 mass spectrometer with moderate sensitivity and scan speed.

Evaluation of reproducibility and quantification performance of three replications in an automated operation is shown in Fig. 2C. Plasma samples from the same batch were processed with three adjacent wells on the plate, and 321, 318, and 319 protein groups were identified by single-shot LC–MS/MS analysis with 60 min gradient, respectively. Among them, 243 proteins were identified in all three replicates, which represents 59.4% of all the identified proteins (Fig. 2C). To assess the precision at the protein level, coefficient of variation (CV) of protein intensity of three replicates were calculated and 85.2% of proteins showing CVs of ≤ 20%, with the median CV% was 9.0% (Fig. 2D). Importantly, more than 91% of the PSMs have missed cleavage number of 0, indicating a good digestion efficiency (Fig. 2E). Figure 2F and Additional file 1: Fig. S1 showed the response correlation of each two replicates was 0.999, 0.998, and 0.998, suggesting that our optimized workflow has a good identification and operation reproducibility (Additional file 1: Fig. S1).

### Performance of the automated plasma proteome profiling procedure

**Fig. 3:**
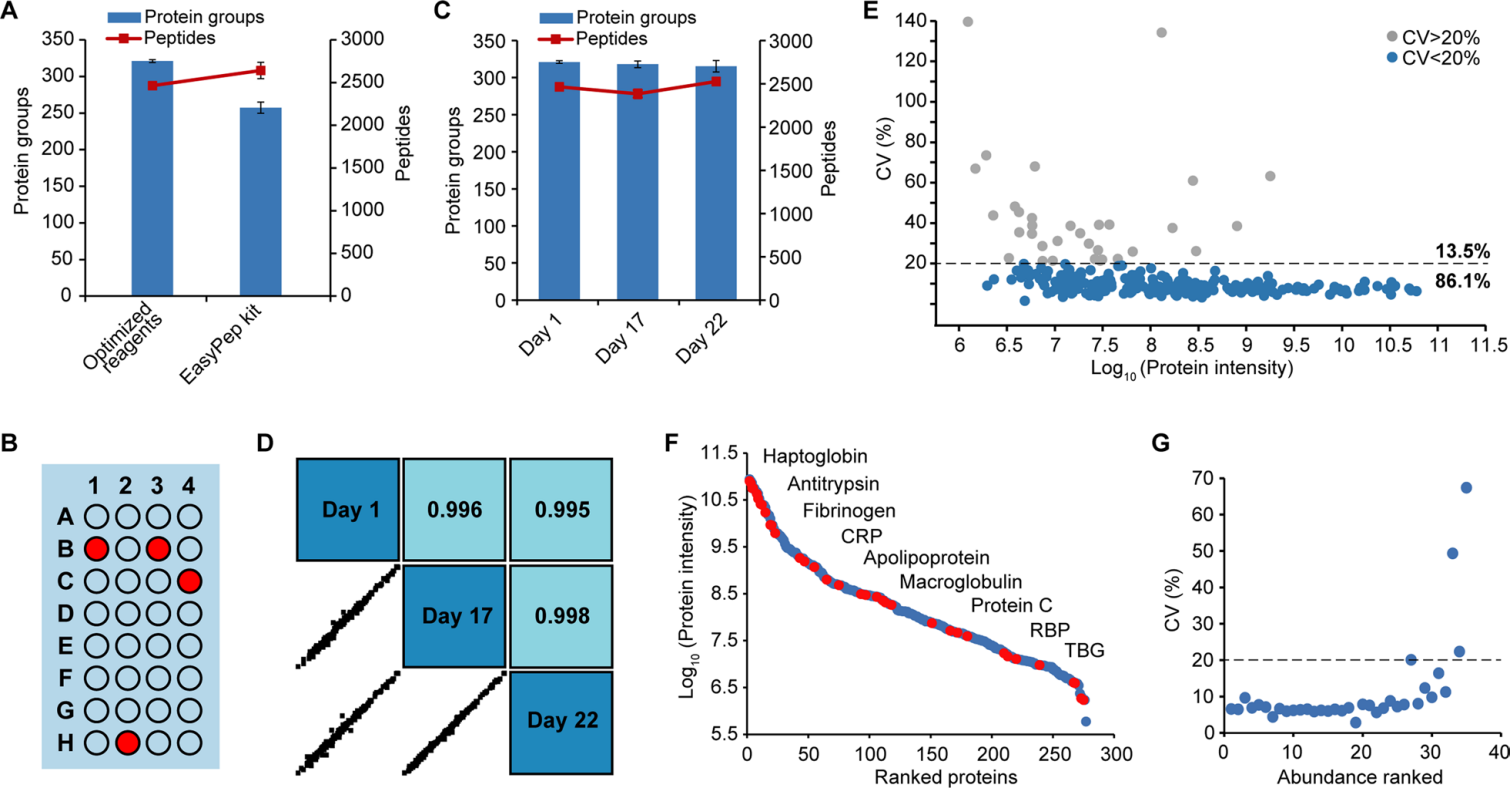
Performance of the automated plasma proteome profiling procedure. **A** Comparison of identified proteins number of AMTK with “Optimized reagents” and “EasyPep reagent kit” (n = 4). **B** Four randomly selected samples from one plate were subjected to micro-flow LC–MS analysis. **C** Average number of protein groups and unique peptides identified of the same sample processed on three different days (day 1, day 17, and day 22) over a period of a month under optimized conditions and subjected to micro-flow LC–MS analysis. Error bars represent standard deviations from three technical replicates. **D** Correlations of the protein intensity between each of the two samples during the three days. **E** CVs of all quantified proteins were calculated for the 4 replicates. Proteins with CV < 20% are colored in blue and those with CV > 20% in gray. **F** The dynamic range of plasma proteome, FDA-approved biomarkers highlighted in red. **G** The CVs of 35 FDA-approved biomarkers from 4 replicates

To test the performance of our home-optimized automated workflow, it was compared with the automated uHTPPP workflow by analyzing the same plasma sample in four replicated wells. According to the official data provided by ThermoFisher in 2020, less than 200 proteins were identified (Application note 65,727). As illustrated in Fig. 3A, an average of 250 proteins were identified when buffers from the EasyPep reagent kit were applied to our own workflow, while our optimized workflow resulted in an average protein identification of 321. It is worth to mention that the official data of EasyPep 96 MS Kit were analyzed using nano-flow LC coupled with Orbitrap HF-X mass spectrometer which should have better performance than Orbitrap Exploris 240 mass spectrometer. These results fully demonstrate the performance of our automated plasma proteomic analysis pipeline.

To further assess the reproducibility of the automated workflow for large-scale clinical sample preparation, we examined both the well-to-well and day-to-day reproducibility. The pipette head of AMTK LH-1808 used in this study has a maximum of 32 channels, hence, four of them were randomly selected for day-to-day repeatability evaluation (Fig. 3B). Automated sample preparation of the same batch of plasma samples was performed on three days (day 1, 17, and 22) over a period of one month. All 12 samples (four per day) were measured by single-shot LC MS analysis with 60 min LC gradient in a single combined analysis sequence. The average value and standard deviations of identified protein groups and unique peptides at each random point were calculated to assess their qualitative stability. Figure 3C shows the average number of protein groups identified per day was 321, 318, and 315. As shown in Fig. 3D, the Pearson correlation coefficients of quantified proteins at one well in these three days were 0.996, 0.995, and 0.998, respectively (Data of other random points were shown in Additional file 1: Fig. S2). CVs of all quantified proteins’ intensity were calculated for the 4 replicates and the median %CV was 8.9%, with 86.1% of proteins showing CVs less than 20% (Fig. 3E).

To evaluate the capability of our workflow in identifying clinically interesting biomarkers, we evaluated the 4 replicates to calculate the CVs and checked the Food and Drug Administration (FDA)-approved biomarkers [24] in our quantified protein list. Among 109 FDA-approved biomarkers, 35 proteins were detected, 31 of them had CVs of less than 20%, and 28 had CVs even less than 10% (Fig. 3F–G). Collectively, the above results indicate that the system has good identification and operation repeatability across replicates of sequentially processed sample in the optimized automated sample preparation system. Its robust performance therefore lays the foundation for its application to high-throughput and long-time continuous analysis of clinical samples.

### Automated plasma proteome profiling of colon cancer

**Fig. 4:**
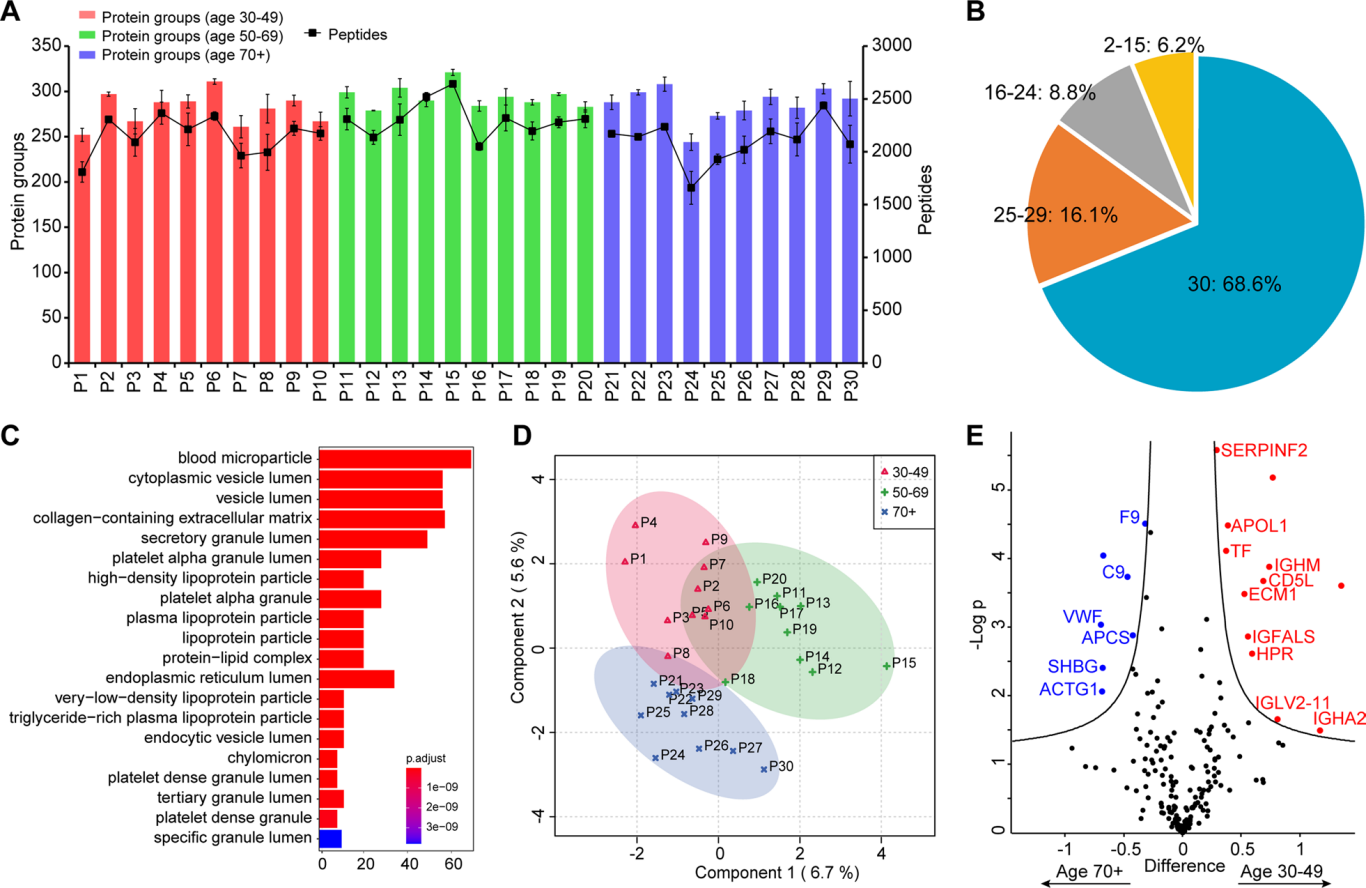
Automated plasma proteome profiling procedure of colon cancer patients. **A** Overview of the identified protein groups and peptides in each individual sample. **B** Percentage of detected proteins in all the 30 samples, in 25–29, in 16–24, in 2–15 samples. **C** Top 20 of Gene Ontology enrichments for cellular component. **D** sPLS-DA scores plots based on the colon cancer patients’ dataset (Red, patients aged 30–49; Green, patients aged 50–69; Blue, patients aged 70 +). **E** Volcano plot of statistical significance against log2-fold change between age 30–49 (n = 10) and age 70 + (n = 10) in colon cancer cohort. Significance is controlled by p-value (independent two-sample *t*-test, two-sided) and minimum fold change (s0 parameter in Perseus) indicated by the cutoff curve

After optimizing the automated plasma proteome profiling workflow, we applied it to the colon cancer plasma proteomic analysis as a proof-of-concept study. A total of 30 colon cancer patient samples at stage III or IV were used for this analysis. According to the age distribution of patients in the sample database, they were divided into three different age groups, including 30–49 years old group, 50–69 years old group, and over 70 years old group, with 10 samples in each group. After processed with our automated plasma proteomics workflow, all 30 samples were analyzed by the micro-flow LC–MS method. On average, 287 protein groups and 2 184 peptides per sample were identified (Fig. 4A). Within the dataset, 68.6% of all proteins (188 protein groups) were identified in all of the 30 samples, 83.5% proteins (228 protein groups) in more than 25 samples, only 3.7% proteins were detected in less than one third of the samples and none of them uniquely detected in one sample (Fig. 4B). The result indicated the high reproducibility and stability of our automated workflow among the large set of plasma samples.

Gene Ontology analysis was performed to annotate the cellular component of plasma proteins (Fig. 4C). The enriched cellular components in the identified plasma proteins mostly involve in extracellular function of the circulatory system, such as blood microparticle, extracellular matrix, and plasma lipoprotein particle, which was consistent with the plasma proteomic reports [25–27].

Sparse partial least-squares discriminant analysis (sPLS-DA) and variable importance in projection (VIP) scores were computed to detect inherent trends within data of all the proteins using their LFQ intensities. As shown in Fig. 4D, the score plot of the sPLS-DA showed separation trends between the three groups, even though the three groups showed considerable overlap, indicating the proteomic differences in colon cancer patients with different ages. The top 34 proteins with the greatest influence on sPLS-DA analysis were showed by heat map (Additional file 1: Fig. S3). We further analyzed potential biomarkers between pairs in all three age groups. As a result, 19 proteins were found differentially expressed between age 30–49 and age 70 + patients (Fig. 4E). There were 11 proteins differentially expressed between age 50–69 and age 70 + patients. In detail, when age 30–49 was compared with age 70 + , 12 proteins were up-regulated, while 7 proteins were down-regulated; when age 50–69 was compared with age 70 + , 4 proteins were up-regulated, while 7 proteins were down-regulated. And only 3 proteins were found up-regulated when age 30–49 was compared with age 50–69 in colon cancer cohort (Additional file 1: Fig. S4). Detailed information of these differentially expressed proteins are provided in Additional file 3: Table S2–S4. Among all these 28 differentially expressed proteins, 24 proteins were in the list of VIP value > 1 in sPLS-DA. The consistency of the results of the two data analysis methods proves that our automated workflow combined micro-flow LC–MS approach is feasible for processing large-scale clinical plasma samples.

## Discussions

Herein, we developed an automated sample preparation and robust analysis platform for proteomic profiling of large-scale clinical plasma samples. Plasma proteins were treated by in solution digestion without protein immunodepletion and prefractionation. Considering the inherent pipetting error of the automated device for minute volumes, we started with 5 μL of plasma samples, which is a small amount for clinical testing, while our manual operation only used a starting amount of 1 μL [15]. Depending on the configuration of the automation platform, tens to hundreds of samples can be processed simultaneously in each working cycle. C18 spintip desalting was performed after automated processing, which is the only step that requires manual handling, but can also be multiplexed on standard centrifuge. As the next step, the automatic sample preparation platform could be combined with the automatic desalting module (which has been commercialized) to achieve full automatization.

From the perspective of data acquisition, the application of micro-flow liquid chromatography in this study improved the analytical throughput and robustness of the plasma samples. Nevertheless, compared with the throughput of the automated pretreatment platform, the throughput of LC–MS analysis still needs to be improved. Systematic studies have shown that the same 1 mm i.d. × 150 mm column could be used to analyze > 7 500 samples including specimens such as human cell lines, tissues, and body fluids and still with excellent reproducibility and protein quantification performance [19]. The results of intra-plate and inter-day repeatability showed that the automated pretreatment platform combined with micro-flow LC–MS had good reproducibility for the analysis of plasma samples. The automated workflow allows quantitative analysis of ~ 300 protein groups in single plasma sample, covering 35 FDA-approved biomarkers. Recently, Blume et al. reported an automated approach for plasma proteomic profiling by using nano-bio interaction properties of multiple engineered magnetic nanoparticles, which could identify more than 2000 proteins from 1 mg of plasma sample [28]. It is therefore important for maintaining a balance between throughput and depth of analysis.

The automatic workflow was further applied to a proof-of-concept plasma proteome study of colon cancer in different age groups. Interestingly, a series of proteins were identified to distinguish different colon cancer age groups, with the most significantly changed proteins found between the two groups with the largest age gap. Over the past several years, multiple studies about aging of the plasma proteome have been conducted. Among the differentially expressed proteins in the three age groups, there are proteins involved in healthy aging, such as IGFALS [29], and proteins that may predict healthy aging and longevity, such as LPA [30] and C9 [31]. In addition, 15 differentially expressed proteins have been reported to have age-dependent changes in the plasma proteome [32, 33]. Surinova et al. detected 88 candidate proteins in the study of non-invasive detection of colorectal cancer based on blood-based markers [34], of which 46 proteins were in our detection list and 4 proteins were in our differentially expressed protein list. However, these significantly changed proteins cannot yet be considered as potential biomarkers for age or colon cancer without data support from non-colon cancer patients and larger cohorts. Because of the high variability of plasma samples between individuals and the influence of factors such as medical history and even mental status, it is necessary to develop generally accepted normalization methods, which will be carried out in our future large-scale sample studies. Nonetheless, the current study demonstrates that our automated workflow is feasible for clinical plasma proteome detection and biomarker analysis.

## Conclusions

In this study, we develop an automated and robust plasma proteomics workflow that can be used for large-scale clinical plasma proteomic analysis. The workflow combines efficient automated sample preparation techniques with micro-flow LC–MS-based methods. Compared with the manual workflow, this approach can analyze dozens of samples simultaneously with higher throughput and reproducibility. Without protein depletion and prefractionation, more than 300 protein groups can be identified in a single analysis, including the quantification of 35 FDA-approved markers from plasma. This workflow is particularly suitable for the detection and analysis of large-scale clinical plasma samples due to its simple and time-saving operation. The results of proof-of-concept study confirmed the feasibility of the workflow to discover potential biomarkers from large clinical plasma sample cohorts.

## Supplementary Information

**Additional file 1:** Supplementary figures of the automated plasma proteome profiling procedure's performance and proteomics analysis of colon cancer patients.

**Additional file 2:** Clinical information of the colon cancer patients.

**Additional file 3:** Significantly changed proteins found in patients.

## Data Availability

The datasets used and/or analyzed during the current study are available from the corresponding author on reasonable request.

## Abbreviations

LC: Liquid chromatography
MS: Mass spectrometry
FDA: Food and Drug Administration
CV: Coefficient of variation
FOBT: Fecal occult blood test
iST: In-StageTip
BCA: Bicinchoninic acid
TCEP: Tris (2-carboxyethyl) phosphine hydrochloride
CAA: 2-Chloroacetamide
TFA: Trifluoroacetic acid
FA: Formic acid
HCD: Higher energy collisional dissociation
PD: Proteome Discoverer
FDR: False discovery rate
PSMs: Peptide spectrum matches
LFQ: Label-free quantification
PEP: Posterior error probability
GO: Gene ontology
uHTPPP: Ultra-high-throughput plasma protein profiling
sPLS-DA: Sparse partial least-squares discriminant analysis
VIP: Variable importance in projection
