# Supplementary material for: Combination of automated sample preparation and micro-flow LC–MS for high-throughput plasma proteomics
Source: Clin Proteomics. 2023 Jan 7;20:3. doi: 10.1186/s12014-022-09390-w (PMC9824974; doi:10.1186/s12014-022-09390-w)
Supplement: Supplementary file 1 — Additional file 1: Supplementary figures of the automated plasma proteome profiling procedure's performance and proteomics analysis of colon cancer patients. [file 12014_2022_9390_MOESM1_ESM.docx]

**SUPPORTING INFORMATION：**Figures S1−S4.


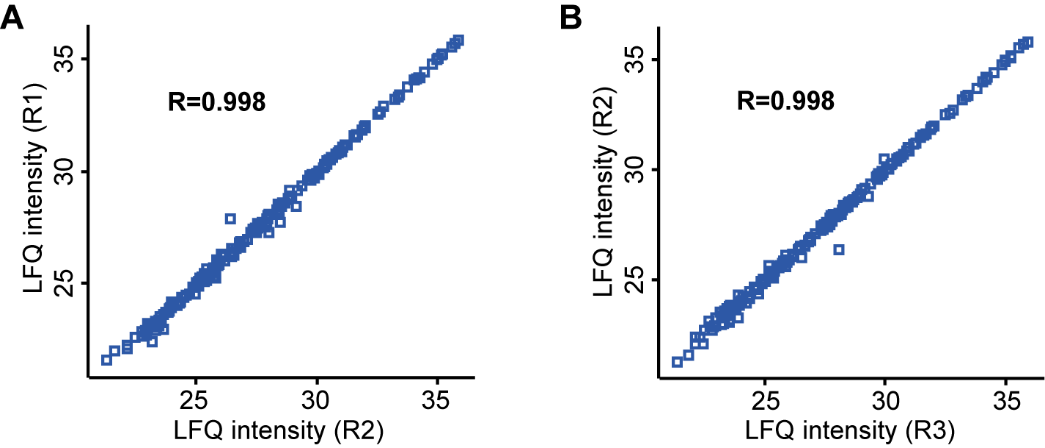


**Figure S1.** (A) Correlation of the protein intensities between replicate 2 and replicate 1.

(B) Correlation of the protein intensities between replicate 3 and replicate 2.


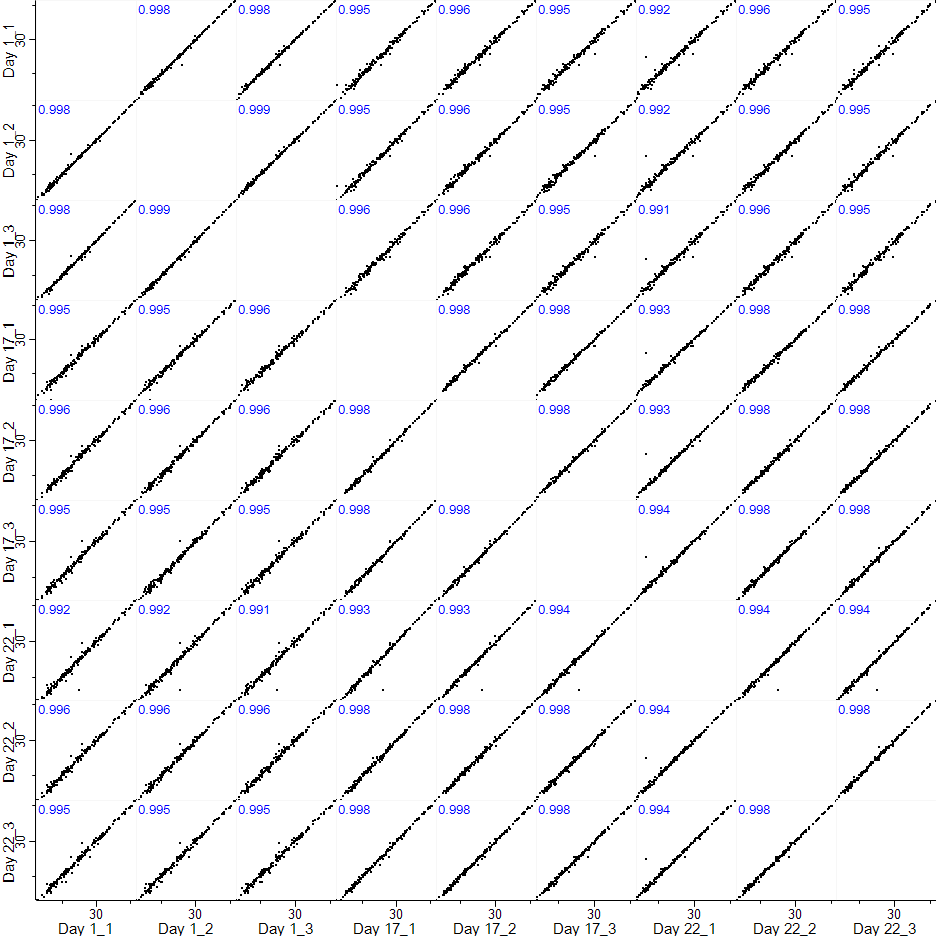


**Figure S2.** Correlations of the protein intensity between any of the two samples during the three days.

**
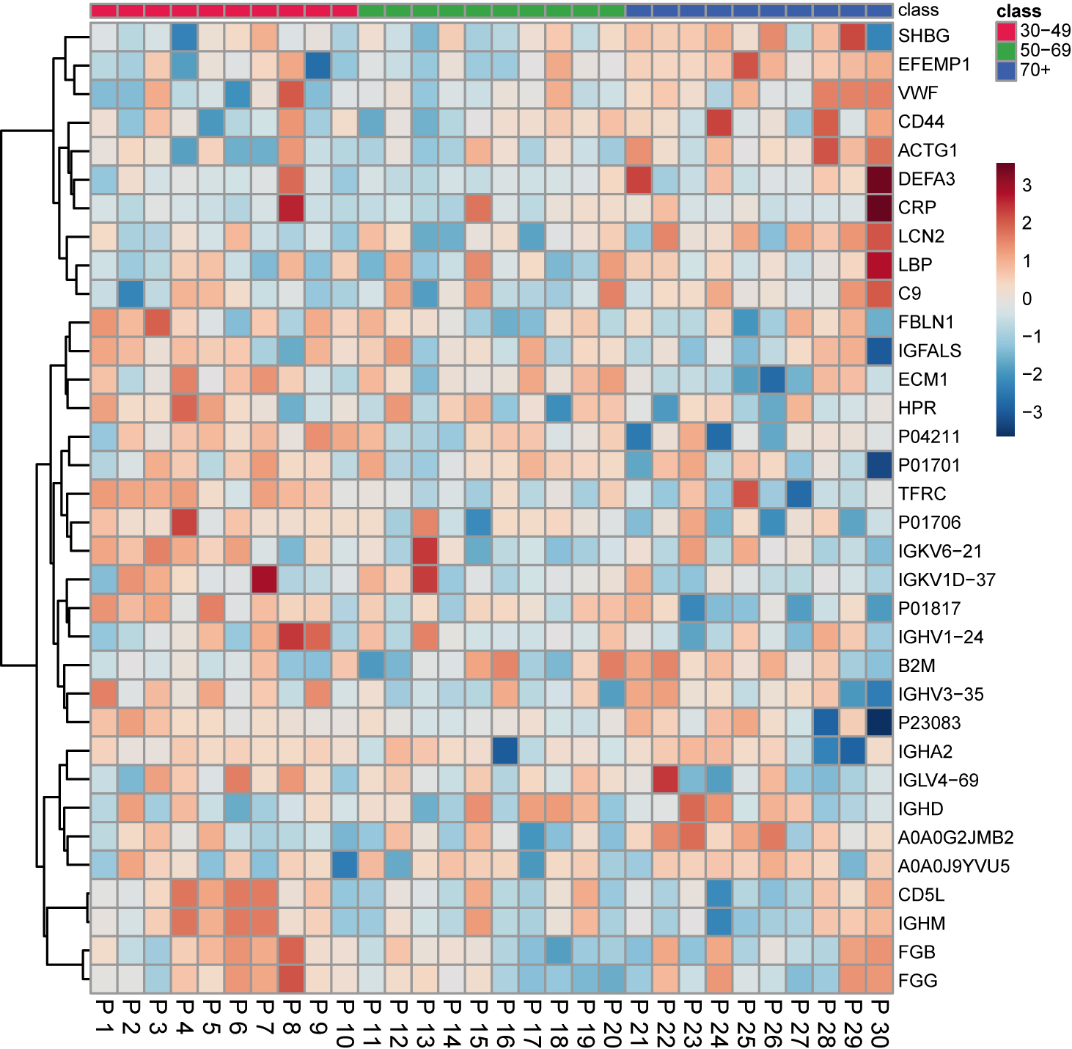
**

**Figure S3.** The top 34 proteins with the greatest influence on PLS-DA analysis were showed by heat map (VIP>1.4).

**
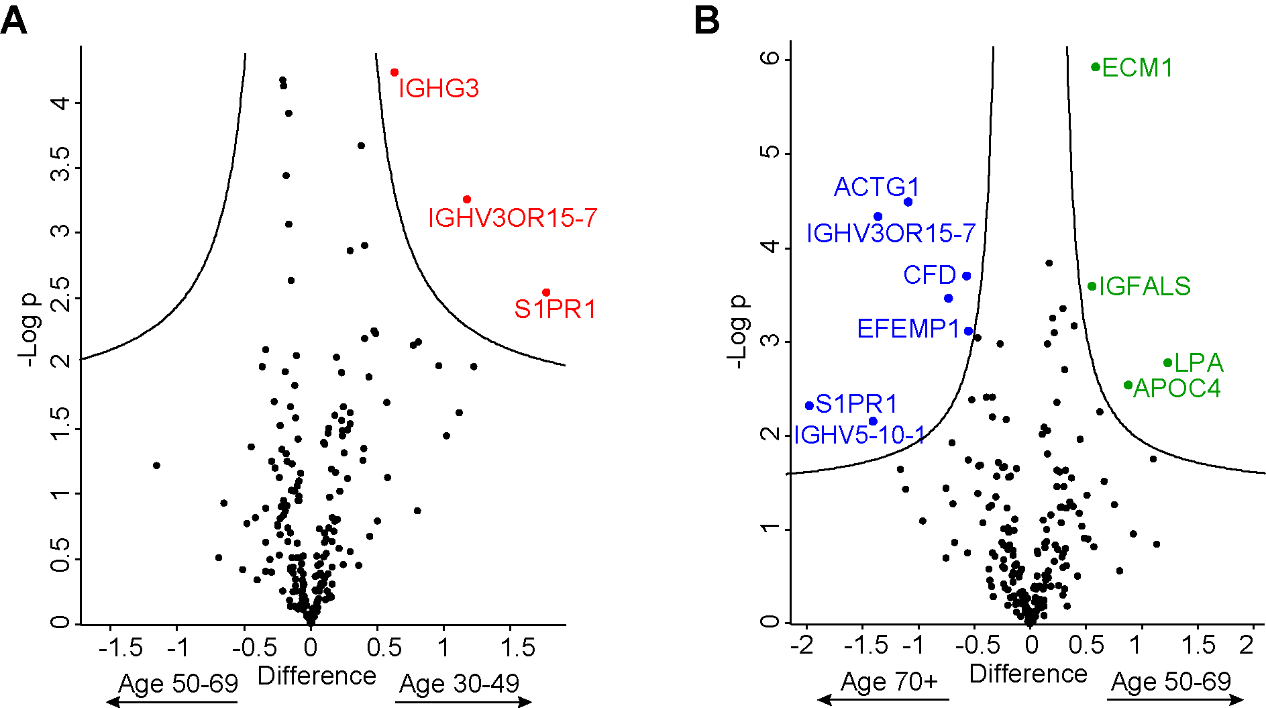
**

**Figure S4.** Volcano plot of statistical significance against log2-fold change between age 30-49 (N = 10), age 50-69 (N = 10) and age >70 (N = 10) in colon cancer cohort. Significance is controlled by p-value (independent two-sample t-test, two-sided) and minimum fold change (s0 parameter in Perseus) indicated by the cutoff curve.
