# Supplementary material for: Combination of automated sample preparation and micro-flow LC–MS for high-throughput plasma proteomics
Source: Clin Proteomics. 2023 Jan 7;20:3. doi: 10.1186/s12014-022-09390-w (PMC9824974; doi:10.1186/s12014-022-09390-w)
Supplement: Supplementary file 2 — Additional file 2: Clinical information of the colon cancer patients. [file 12014_2022_9390_MOESM2_ESM.docx]

**SUPPORTING INFORMATION：**Table S1.

**Table S1.** The clinical information of patients with colon cancer

| Case | Sex | Age | Clinical stage | TNM |
| --- | --- | --- | --- | --- |
| P 1 | M | 34 | IIIB | pT3N2M0 |
| P 2 | M | 30 | IV | pT3N1M1 |
| P 3 | M | 41 | IIIB | pT3N1M0 |
| P 4 | M | 46 | IIIB | pT3N2aM0 |
| P 5 | M | 43 | IVA | pT3aN2bM1a |
| P 6 | F | 35 | IV | pT3N0M1 |
| P 7 | F | 34 | IV | T3N2M1 |
| P 8 | F | 37 | IV | TxNxM1 |
| P 9 | F | 47 | ⅢA | pT4aN2M0 |
| P 10 | F | 45 | III | pT1N1M0 |
| P 11 | M | 53 | IIIA | pT2N1M0 |
| P 12 | M | 58 | ⅢC | pT4N2M0 |
| P 13 | M | 51 | IV | pT4aN3M1 |
| P 14 | M | 63 | IV | TxNxM1 |
| P 15 | M | 65 | IV | pT3N2M1 |
| P 16 | F | 58 | IV | pT4N3M1 |
| P 17 | F | 57 | ⅢB | pT3N2M0 |
| P 18 | F | 56 | III | pT3N2aM0 |
| P 19 | F | 65 | III | pT3N1M0 |
| P 20 | F | 66 | IV | pT4bN1M1 |
| P 21 | M | 72 | ⅢA | pT3N1M0 |
| P 22 | M | 80 | IV | pT4aN1M1 |
| P 23 | M | 73 | IV | TxNxM1 |
| P 24 | M | 76 | IV | TxNxM1 |
| P 25 | M | 74 | IV | T3N2M1 |
| P 26 | F | 76 | IV | TxNxM1 |
| P 27 | F | 73 | IV | TxNxM1 |
| P 28 | F | 72 | IV | TxNxM1 |
| P 29 | F | 72 | IV | TxNxM1 |
| P 30 | F | 79 | IV | TxNxM1 |

TNM: Tumor Node metastasis.

x: missing information (No surgical information for patients with distal metastases)
