# Supplementary material for: Combination of automated sample preparation and micro-flow LC–MS for high-throughput plasma proteomics
Source: Clin Proteomics. 2023 Jan 7;20:3. doi: 10.1186/s12014-022-09390-w (PMC9824974; doi:10.1186/s12014-022-09390-w)
Supplement: Supplementary file 3 — Additional file 3: Significantly changed proteins found in patients. [file 12014_2022_9390_MOESM3_ESM.docx]

**SUPPORTING INFORMATION：**Tables S2−S4.

**Table S2.** Proteins differentially expressed between age 30-49 and age 70+ patients

| **-Log(P-value)** | **Fold change** | **Majority protein IDs** | **Gene names** | **Protein names** |
| --- | --- | --- | --- | --- |
| 5.57 | 0.3 | P08697 | SERPINF2 | Alpha-2-antiplasmin |
| 5.18 | 0.8 | P01817 |  | Ig heavy chain V-II region MCE |
| 4.50 | -0.3 | P00740 | F9 | Coagulation factor IX |
| 4.48 | 0.4 | O14791 | APOL1 | Apolipoprotein L1 |
| 4.11 | 0.4 | P02787 | TF | Serotransferrin |
| 4.04 | -0.7 | A0A0G2JMB2 |  |  |
| 3.88 | 0.7 | P01871 | IGHM | Ig mu chain C region |
| 3.73 | -0.5 | P02748 | C9 | Complement component C9 |
| 3.67 | 0.7 | O43866 | CD5L | CD5 antigen-like |
| 3.60 | 1.4 | P04211 |  | Ig lambda chain V region 4A |
| 3.48 | 0.5 | Q16610 | ECM1 | Extracellular matrix protein 1 |
| 3.03 | -0.7 | P04275 | VWF | von Willebrand factor |
| 2.88 | -0.4 | P02743 | APCS | Serum amyloid P-component |
| 2.86 | 0.6 | P35858 | IGFALS | Insulin-like growth factor-binding protein complex acid labile subunit |
| 2.61 | 0.6 | P00739 | HPR | Haptoglobin-related protein |
| 2.41 | -0.7 | I3L145 | SHBG | Sex hormone-binding globulin |
| 2.06 | -0.7 | A0A2R8Y793 | ACTG1 | Actin |
| 1.66 | 0.8 | P01706 | IGLV2-11 | Ig lambda chain V-II region BOH |
| 1.49 | 1.2 | P01877 | IGHA2 | Ig alpha-2 chain C region |

**Table S3.** Proteins differentially expressed between age 50-69 and age 70+ patients

| **-Log(P-value)** | **Fold change** | **Majority protein IDs** | **Gene names** | **Protein names** |
| --- | --- | --- | --- | --- |
| 5.93 | 0.6 | Q16610 | ECM1 | Extracellular matrix protein 1 |
| 4.49 | -1.1 | A0A2R8Y793 | ACTG1 | Actin |
| 4.34 | -1.4 | A0A075B7D8 | IGHV3OR15-7 |  |
| 3.70 | -0.6 | K7ERG9 | CFD | Complement factor D |
| 3.59 | 0.6 | P35858 | IGFALS | Insulin-like growth factor-binding protein complex acid labile subunit |
| 3.47 | -0.7 | A0A0G2JMB2 |  |  |
| 3.11 | -0.5 | Q12805 | EFEMP1 | EGF-containing fibulin-like extracellular matrix protein 1 |
| 2.78 | 1.2 | P08519 | LPA | Apolipoprotein(a) |
| 2.54 | 0.9 | P55056 | APOC4 | Apolipoprotein C-IV |
| 2.32 | -2.0 | P21453 | S1PR1 | Sphingosine 1-phosphate receptor 1 |
| 2.15 | -1.4 | A0A0J9YXX1 | IGHV5-10-1 |  |

**Table S4.** Proteins differentially expressed between age 30-49 and age 50-69 patients

| **-Log(P-value)** | **Fold change** | **Majority protein IDs** | **Gene names** | **Protein names** |
| --- | --- | --- | --- | --- |
| 4.23 | 0.6 | A0A4W9A917 | IGHG3 | Ig gamma-3 chain C region |
| 3.26 | 1.2 | A0A075B7D8 | IGHV3OR15-7 |  |
| 2.54 | 1.8 | P21453 | S1PR1 | Sphingosine 1-phosphate receptor 1 |

The p value was calculated using Student’s *t*-test. Fold change with positive value demonstrates up-regulated of protein expression, and negative value indicates down-regulated of protein expression.
